# Early versus late clinical outcomes following same day discharge after elective percutaneous coronary intervention: A systematic review and meta-analysis

**DOI:** 10.1097/MD.0000000000014025

**Published:** 2019-01-04

**Authors:** Hongtao Lu, Wenjun Guan, Yanhua Zhou, Hong Bao

**Affiliations:** a Jingzhou Central Hospital of Cardiology, The Second Clinical Medical College, Yangtze University, Jingzhou; b Jingzhou First People's Hospital, the First Clinical Medical College; c Jiangling County People's Hospital of Cardiology, Jingzhou, Hubei, People's Republic of China.

**Keywords:** clinical outcomes, elective percutaneous coronary intervention, major adverse cardiac events, re-hospitalization, same day discharge

## Abstract

**Background:**

Nowadays 57% of the cardiologists based in the United Kingdom and 32% of the cardiologists based in Canada utilize same day discharge (SDD) following elective percutaneous coronary intervention (PCI) as a routine practice. In this analysis, we aimed to systematically assess early versus late clinical outcomes following SDD after elective PCI.

**Methods:**

The Medical Literature Analysis and Retrieval System Online, the Cochrane Central, the Resources from the United States National Library of Medicine (www.ClinicalTrials.gov: http://www.clinicaltrials.gov) and EMBASE were carefully searched for relevant English publications which reported early versus late clinical outcomes in patients who were discharged on the same day following revascularization by PCI. Relevant clinical outcomes which were reported in the original studies were considered as the endpoints in this analysis. Odd ratios (OR) and 95% confidence intervals (CI) were used to represent the data, and RevMan 5.3 was used as the statistical software.

**Results:**

A total number of 21, 687 participants (enrollment time period from the year 1998 to the year 2015) were assigned to this analysis. When early versus late clinical outcomes were compared in patients who were discharged on the same day following elective PCI, major adverse cardiac events (OR: 0.75, 95% CI: 0.31–1.79; *P* = .51), mortality (OR: 0.26, 95% CI: 0.06–1.06; *P* = .06), stroke (OR: 1.46, 95% CI: 0.72–2.94; *P* = .29), arrhythmia (OR: 1.30, 95% CI: 0.64–2.63; *P* = .47), hematoma (OR: 1.00, 95% CI: 0.60–1.66; *P* = 1.00) and major bleeding from access site (OR: 1.68, 95% CI: 0.22–12.85; *P* = .62) were not significantly different. Post-procedural myocardial infarction (OR: 2.01, 95% CI: 0.71–5.70; *P* = .19) and minor bleeding from access site (OR: 6.61, 95% CI: 0.86–50.66; *P* = .07) were also similarly manifested. However, re-hospitalization was significantly higher in those patients with late clinical outcomes (OR: 0.18, 95% CI: 0.07–0.44; *P* = .0002).

**Conclusions:**

In those patients who were discharged from the hospital on the same day following elective PCI, no significant difference was observed in the assessed early versus late clinical outcomes. However, late clinical outcomes resulted in a significantly higher rate of re-hospitalization. Larger studies should confirm this hypothesis.

## 1 Introduction

Cardiovascular disease (CVD) is among the major causes of mortality in this new era.^[1]^ Percutaneous coronary intervention (PCI) remains the most common option for majority of the patients (accounting for about 3.6% of all operating theatres in the United States^[2]^) and an approximate total number of 500, 000 procedures are carried out annually in the United States.^[3]^ Following this invasive procedure, patients are observed for at least 24 hours before discharge from the hospital in order to prevent any post-procedural complication. However, with advanced development in Interventional cardiology including newer intra-procedural management guidelines, and considering the high daily hospital costs, and the total number of patients opting for this revascularization strategy requiring places to accommodate new patients, elective PCI on an outpatient basis for patients with stable coronary artery disease (CAD) has recently shown to be safe.^[4]^ Same day discharge (SDD) following elective PCI was successfully being carried out in several PCI capable centers across the globe.^[5–6]^

Even if PCI practice has evolved resulting in a decline in the rate of post-procedural complications, hospitals and institutions might still not take the risk to implement SDD following this invasive procedure in fear of unexpected unwanted complications as well as the unknown adverse events associated with this SDD instead of an overnight stay to watch for any complication.

SOCRATES (Study of Costs Realized After Percutaneous Coronary Intervention Employing Same Day Discharge) recently randomized patients for the study of SDD following elective PCI,^[7]^ but unfortunately the study was terminated due to a lack of participants. However, a recent meta-analysis demonstrated similar clinical outcomes in patients who were discharged on the same day versus those patients who stayed overnight in the hospital post PCI.^[8]^

When considering SDD following PCI from the point of view of a physician, it was also necessary to consider it from the point of view of a patient. Many patients prefer recovering at home following this invasive procedure for various reasons including comfort, lower hospital cost, and other similar facilities.^[4]^ Therefore, nowadays, 57% of the cardiologists based in the United Kingdom and 32% of the cardiologists based in Canada utilize SDD as a routine practice.^[9]^ However, there is not enough evidence to support early and late clinical outcomes of SDD following coronary angioplasty.

In this analysis, we aimed to systematically assess early versus late clinical outcomes following SDD after elective PCI.

## 2 Methods

### 2.1 Search databases and search terms

MEDLARS (Medical Literature Analysis and Retrieval System Online), Cochrane Central, Resources from the United States National Library of Medicine (www.ClinicalTrials.gov: http://www.clinicaltrials.gov) and EMBASE were carefully searched with reference to the PRISMA study guideline,^[10]^ for relevant English publications comparing early versus late clinical outcomes in patients who were discharged on the same day following revascularization by elective PCI.

The following search terms were used:

- “same day discharge and percutaneous coronary intervention”;
- “same day discharge and PCI”;
- “same day discharge and coronary angioplasty”;
- “same day discharge and coronary intervention”;
- “same day discharge and ambulatory”;
- “same day discharge and PCI and clinical outcomes”;
- “same day discharge and coronary artery intervention”;
- “early discharge and percutaneous coronary intervention”.

All the search databases were used to retrieve relevant publications using the above-mentioned search terms.

### 2.2 Inclusion and exclusion criteria

Studies were included if:

- They were randomized or observational cohorts/registries/retrospective studies comparing early versus late clinical outcomes in patients who were discharged on the same day following PCI;
- They consisted of patients with elective PCI.

Studies were excluded based on the following criteria:

- Either early or late clinical outcomes were not reported;
- They consisted of patients who were not discharged on the same day following PCI;
- They did not report similar outcomes for early and late follow-up time periods;
- They consisted of data which could not be used in this analysis;
- They were duplicated studies.

### 2.3 Types of participants, outcomes reported and follow-up time periods

All the participants were candidates for elective PCI who were discharged on the same day following this interventional procedure.

The clinical outcomes which were analyzed included:

- Major adverse cardiac events (MACEs) consisting of death, myocardial infarction, and repeated revascularization;
- Mortality;
- Post-procedural myocardial infarction (MI);
- Stroke;
- Arrhythmia;
- Major bleeding from access site;
- Minor bleeding from access site;
- Hematoma;
- Re-hospitalization.

Patients who were assigned to the early clinical outcome group had a mean follow-up time period ranging from hours after the procedure to 7 days post-procedure (with the exception of 1 study which had an early follow-up of 30 days).

Patients who were assigned to the late clinical outcome group had a mean follow-up time period ranging from over 24 hours to 30 days (with the exception of 1 study which had a late follow-up time period of 1 year).

The types of participants, outcomes which were assessed and the respective follow-up time periods have been reported in Table 1.

**Table 1:**
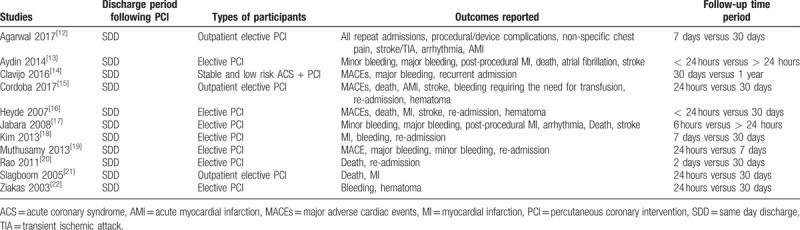
Types of participants, outcomes reported, discharge period, follow-up time periods.

### 2.4 Data extraction and quality assessment

The total number of participants who were discharged on the same day following PCI, the total number of events, the respective clinical outcomes, the time period of patients’ enrollment, and data referring to the baseline features of the participants were carefully extracted and checked by 4 independent reviewers. Any disagreement which followed was resolved by consensus.

The methodological quality of the trials was assessed with respect to the criteria suggested by the Cochrane Collaboration.^[11]^

### 2.5 Statistical analysis

The latest version of the RevMan software (version 5.3) was used to carry out the statistical analysis. Odds ratios (OR) and 95% confidence intervals (CI) were generated to represent the data following the subgroup analysis.

Heterogeneity was assessed by the Q statistic and the I^2^ statistic tests respectively. During the subgroup analysis, a *P* value less or equal to .05 was considered statistically significant. When the I^2^ value was used to assess heterogeneity, an increasing value of I^2^ indicated an increased level of heterogeneity.

A fixed effects (I^2^ <50%) statistical model or a random effects (I^2^ >50%) statistical model was used based on the I^2^ value which was obtained.

Sensitivity analysis was carried out using an exclusion method, and publication bias were assessed using funnel plots.

### 2.6 Ethical approval

Ethical or board review approval was not required for this type of study.

## 3 Results

### 3.1 Search outcomes

A total number of 396 publications were obtained through search databases. The 4 reviewers carefully assessed the titles and abstract and publications which were irrelevant were directly eliminated (345 articles).

Fifty-one (51) full-text articles were assessed for eligibility.

Another careful assessment of the full-text articles was carried out and further irrelevant articles were eliminated: meta-analysis (3), case studies (5), literature reviews (3), letters to editors (4), control group was absent (8), corresponding endpoints were not reported (2), repeated studies (15).

**Figure 1:**
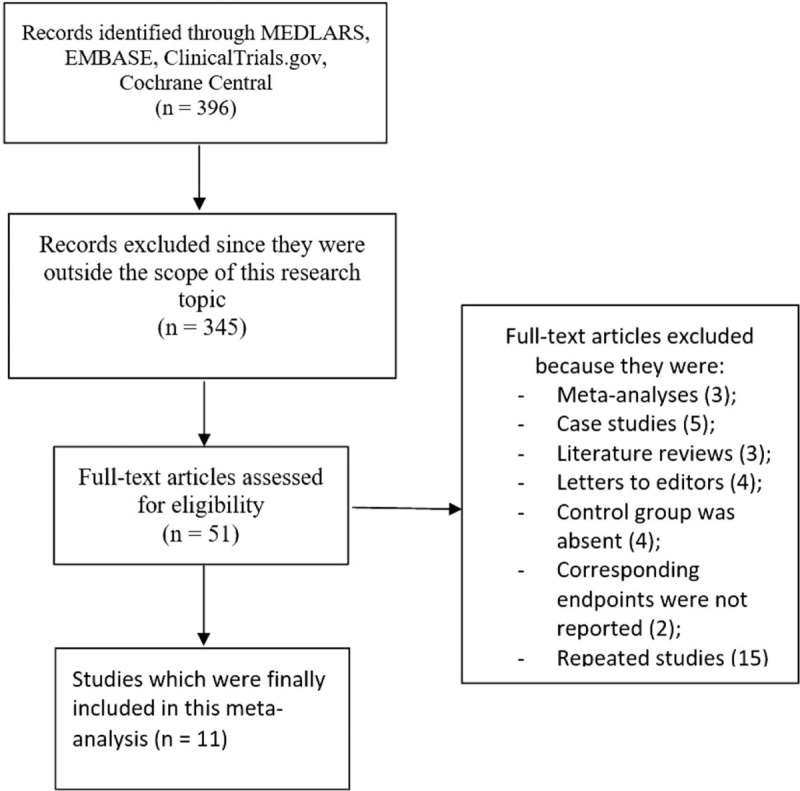
Flow diagram representing the study selection.

Finally, 11 articles^[12–22]^ were selected to be included in this analysis as shown in Figure 1.

### 3.2 Main features of the studies

The main features of the studies have been listed in Table 2.

**Table 2:**
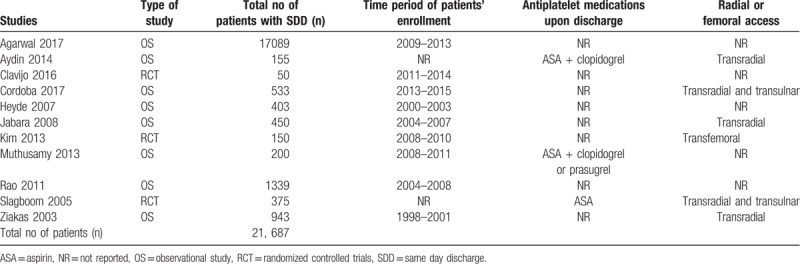
Main features of the studies.

A total number of 21, 687 participants (enrollment time period from the year 1998 to the year 2015) were assigned to this analysis. Three studies were randomized trials whereas the remaining 8 studies were observational cohorts. Most of the patients underwent re-vascularization by the transradial approach and aspirin + clopidogrel were the main anti-platelet agents which were continually being used after the procedure.

### 3.3 Baseline characteristics of the participants

The baseline characteristics of the participants have been listed in Table 3.

**Table 3:**
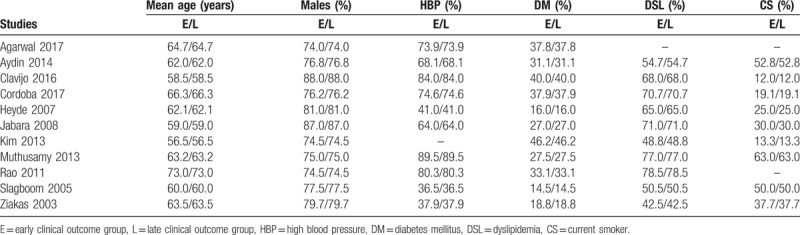
Baseline features of the studies.

The participants were mainly male patients (74.0–88.0) % with a mean age of (56.5–73.0) years as shown in Table 3. Other features including the several cardiovascular risk factors (hypertension, diabetes mellitus, dyslipidemia, and current smoking) have also been listed in the Table. Overall, there was no significant difference in baseline features reported between the participants who were assigned to the early versus the late follow-up groups.

### 3.4 Main results of this analysis

**Figure 2:**
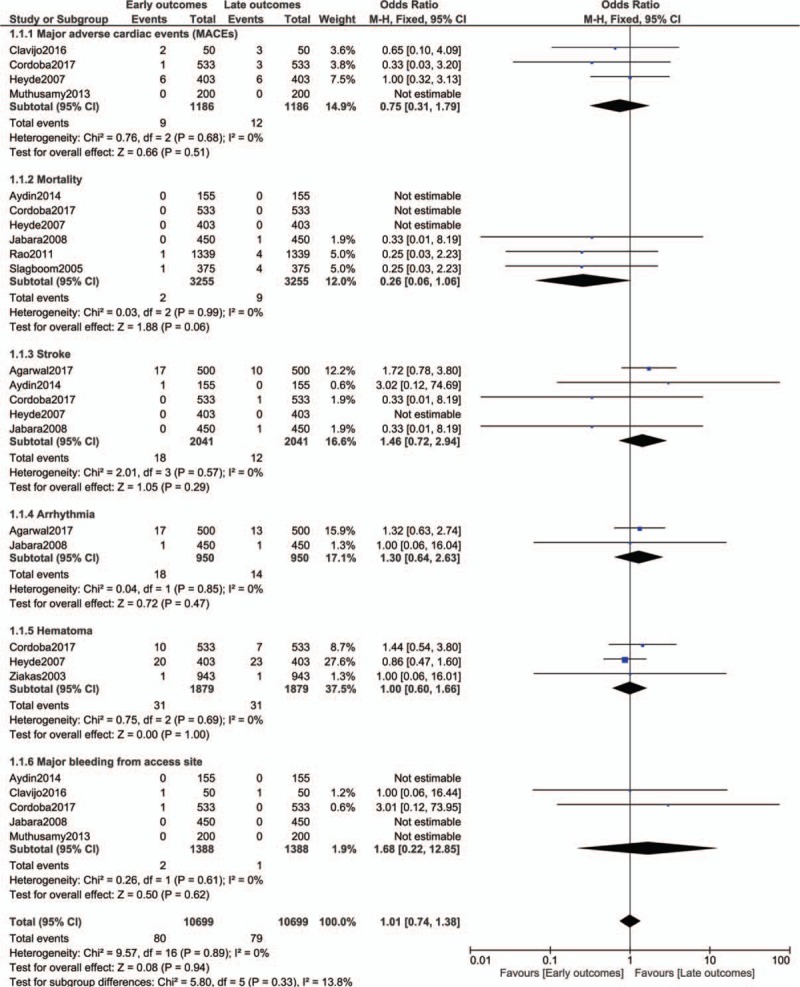
Early versus late clinical outcomes observed in patients who were discharged on the same day following coronary angioplasty (Part 1).

When early versus late clinical outcomes were compared in patients who were discharged on the same day following PCI, MACEs (OR: 0.75, 95% CI: 0.31–1.79; *P* = .51), mortality (OR: 0.26, 95% CI: 0.06–1.06; *P* = .06), stroke (OR: 1.46, 95% CI: 0.72–2.94; *P* = .29), arrhythmia (OR: 1.30, 95% CI: 0.64–2.63; *P* = .47), hematoma (OR: 1.00, 95% CI: 0.60–1.66; *P* = 1.00) and major bleeding from access site (OR: 1.68, 95% CI: 0.22–12.85; *P* = .62) were not significantly different as shown in Figure 2.

**Figure 3:**
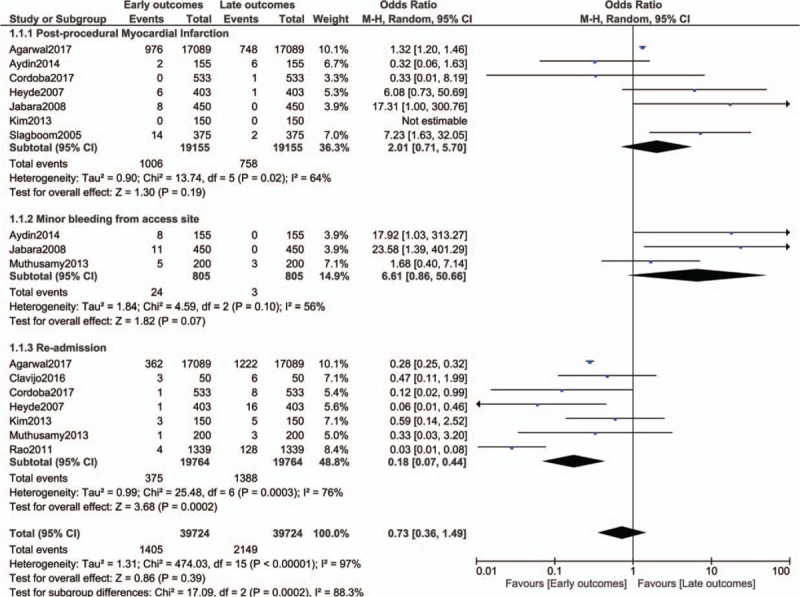
Early versus late clinical outcomes observed in patients who were discharged on the same day following coronary angioplasty (Part 2).

Post-procedural MI (OR: 2.01, 95% CI: 0.71–5.70; *P* = .19) and minor bleeding from access site (OR: 6.61, 95% CI: 0.86–50.66; *P* = .07) were also similarly manifested as shown in Figure 3. However, re-hospitalization was significantly higher in those patients with late clinical outcomes (OR: 0.18, 95% CI: 0.07–0.44; *P* = .0002) as shown in Figure 3.

The main results of this analysis have been summarized in Table 4.

**Table 4:**
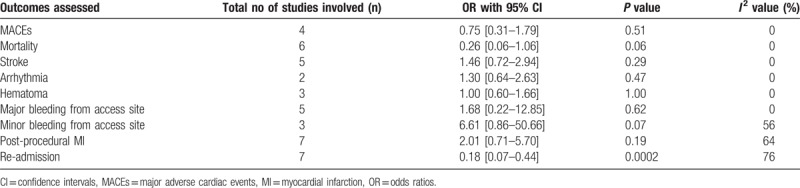
Results of this analysis.

### 3.5 Sensitivity analysis and publication bias

Consistent results were obtained throughout. Even when the study with the largest number of patients was excluded, no significant difference in results was observed. Post-procedural MI (OR: 2.45, 95% CI: 0.47–12.72; *P* = .29), and re-hospitalization (OR: 0.16, 95% CI: 0.04–0.59; *P* = .006) did not significantly differ as compared to the main results.

**Figure 4:**
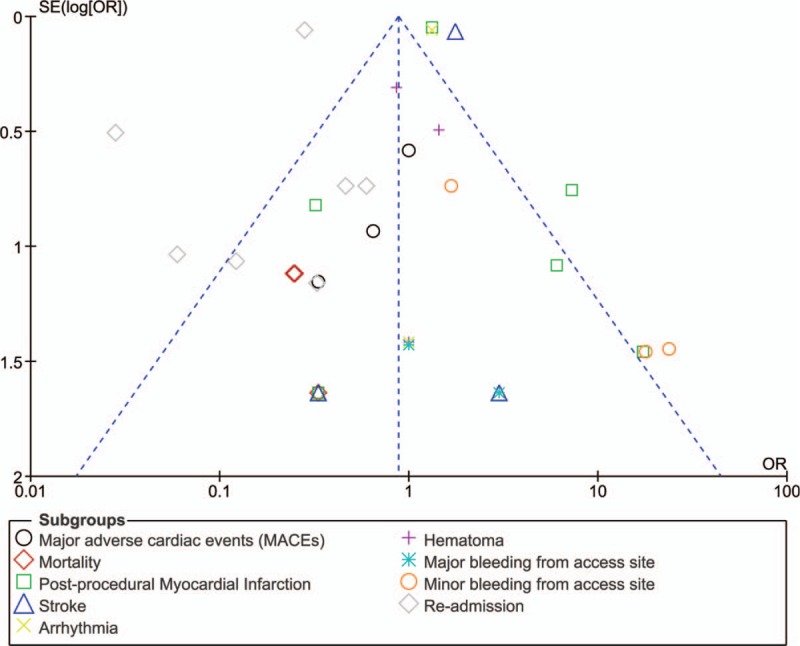
Funnel plot showing publication bias (A).

**Figure 5:**
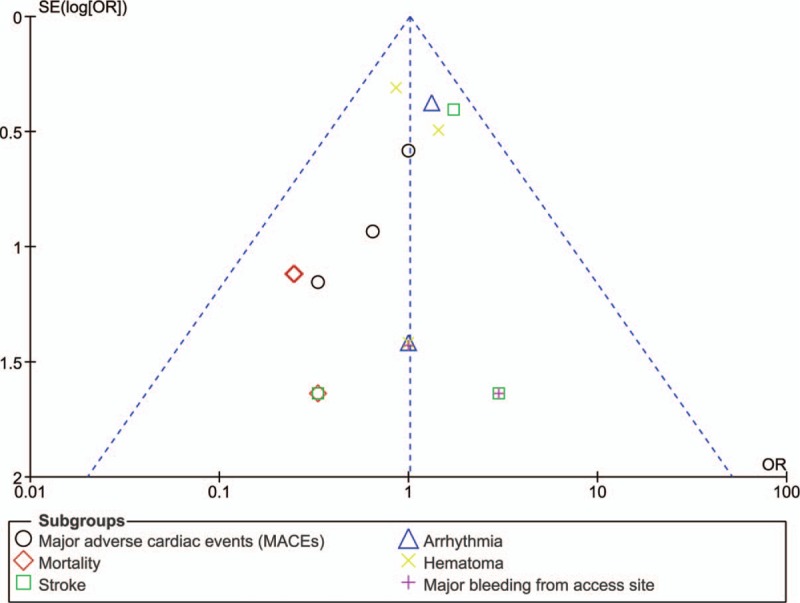
Funnel plot showing publication bias (B).

By assessing the funnel plots which were generated from the RevMan software, only low evidence of publication bias was observed among all the studies that assessed the events reported in early versus late clinical outcomes following SDD after PCI as shown in Figures 4 and 5.

## 4 Discussion

Our analysis comparing early versus late clinical outcomes in patients who were discharged on the same day following PCI showed no significant difference between the 2 groups based on the outcomes that were assessed. However, re-hospitalization was significantly higher in those patients with late clinical outcomes after PCI.

A meta-analysis which compared SDD versus overnight stay in the hospital following PCI showed the former not to be associated with major complications, and the authors stated that SDD appeared safe in selected patients undergoing elective PCI.^[23]^ Moreover, even if the femoral access was more delicate in comparison to the radial or ulnar access for intervention, a retrospective study which was carried out with participants assigned to elective PCI at the Mount Sinai Hospital in New York, showed that if the respective protocol was correctly followed, SDD was completely safe in uncomplicated elective PCI via the femoral access.^[24]^

Eleven hundred ninety elective PCI were retrospectively reviewed at the Red Cross General Hospital to assess for the feasibility and safety of SDD for selected patients undergoing complex PCI using the forearm approach. The authors concluded that SDD was safe in selected patients without any complication.^[25]^

In a recent study which evaluated time trend in SDD to compare certain clinical outcomes including mortality, bleeding and acute kidney injury following contrast injection during the procedure, and which evaluated patients’ satisfaction and patients’ hospital costs for SDD versus overnight stay following the invasive procedure, the authors concluded that with a patient-centered approach, SDD increased tremendously with a safety success rate of over 75% of all the patients who underwent elective PCI.^[4]^ The authors also stated that the patients were very satisfied with the lower hospital cost following this SDD following coronary angioplasty. This strategy should be beneficial to health cost in the future.^[5]^

Now that we know SDD was safe in selected patients following elective PCI, our analysis showed no significant difference with respect to the early versus late clinical outcome. However, re-hospitalization was significantly due to late clinical outcomes and further workups should be carried out on this particular aspect.

## 5 Limitations

Limitations were as follow: first of all, the early and late time period varied from study to study. Not all the study reported post-interventional outcomes during the same follow-up time period. Therefore, even if this is not a major problem, it might be considered as a minor limitation of this analysis. However, in order to resolve this limitation, 4 studies with the same early and late follow-up time periods were also compared and a result similar to the main analysis was obtained. MACEs (OR: 0.78, 95% CI: 0.29–2.09; *P* = .62), re-hospitalization (OR: 0.08, 95% CI: 0.02–0.34; *P* = .0007) and results for the other outcomes were not significantly different with reference to the results of the main analysis. Secondly, due to the inclusion of several observational studies might have introduced bias and could be another limitation of this analysis. In addition, another limitation might be the fact that adverse clinical outcomes could have also been due to anti-platelet agent non-compliance which was not reported in the original study. Even anti-platelet agents which were used by the participant's post PCI were not stated in some of the original studies. This might have had an influence on the main results. At last, even if the total number of participants was enough to reach a robust conclusion, an even larger number of participants might have been more advantageous.

## 6 Conclusions

In those patients who were discharged from the hospital on the same day following elective PCI, no significant difference was observed in the assessed early versus late clinical outcomes. However, late clinical outcomes resulted in a significantly higher rate of re-hospitalization. Larger studies should confirm this hypothesis.

## Author contributions

HL, WG, YZ, and BH were responsible for the conception and design, acquisition of data, analysis and interpretation of data, drafting the initial manuscript and revising it critically for important intellectual content. HL and WG contributed equally as first co-authors and wrote the final draft. All the authors approved the manuscript as it is.

**Conceptualization:** Hongtao Lu, Wenjun Guan, Hong Bao.

**Data curation:** Hongtao Lu, Wenjun Guan, Yanhua Zhou, Hong Bao.

**Formal analysis:** Hongtao Lu, Wenjun Guan, Yanhua Zhou, Hong Bao.

**Funding acquisition:** Hongtao Lu, Wenjun Guan, Yanhua Zhou, Hong Bao.

**Investigation:** Hongtao Lu, Wenjun Guan, Yanhua Zhou, Hong Bao.

**Methodology:** Hongtao Lu, Wenjun Guan, Yanhua Zhou, Hong Bao.

**Project administration:** Hongtao Lu, Wenjun Guan, Yanhua Zhou, Hong Bao.

**Resources:** Hongtao Lu, Wenjun Guan, Yanhua Zhou, Hong Bao.

**Software:** Hongtao Lu, Wenjun Guan, Yanhua Zhou, Hong Bao.

**Supervision:** Hongtao Lu, Wenjun Guan, Yanhua Zhou, Hong Bao.

**Validation:** Hongtao Lu, Wenjun Guan, Yanhua Zhou, Hong Bao.

**Visualization:** Hongtao Lu, Wenjun Guan, Yanhua Zhou, Hong Bao.

**Writing – original draft:** Hongtao Lu, Wenjun Guan, Hong Bao.

**Writing – review & editing:** Hongtao Lu, Wenjun Guan, Hong Bao.
